# Using the Machine Vision Method to Develop an On-machine Insert Condition Monitoring System for Computer Numerical Control Turning Machine Tools

**DOI:** 10.3390/ma11101977

**Published:** 2018-10-14

**Authors:** Wei-Heng Sun, Syh-Shiuh Yeh

**Affiliations:** 1 Institution of Mechatronic Engineering, National Taipei University of Technology, Taipei 10608, Taiwan; sun245689@gmail.com; 2 Department of Mechanical Engineering, National Taipei University of Technology, Taipei 10608, Taiwan

**Keywords:** machine vision, on-machine monitoring, tool insert condition, computer numerical control, turning machine tools

## Abstract

This study uses the machine vision method to develop an on-machine turning tool insert condition monitoring system for tool condition monitoring in the cutting processes of computer numerical control (CNC) machines. The system can identify four external turning tool insert conditions, namely fracture, built-up edge (BUE), chipping, and flank wear. This study also designs a visual inspection system for the tip of an insert using the surrounding light source and fill-light, which can be mounted on the turning machine tool, to overcome the environmental effect on the captured insert image for subsequent image processing. During image capture, the intensity of the light source changes to ensure that the test insert has appropriate surface and tip features. This study implements outer profile construction, insert status region capture, insert wear region judgment, and calculation to monitor and classify insert conditions. The insert image is then trimmed according to the vertical flank, horizontal blade, and vertical blade lines. The image of the insert-wear region is captured to monitor flank or chipping wear using grayscale value histogram. The amount of wear is calculated using the wear region image as the evaluation index to judge normal wear or over-wear conditions. On-machine insert condition monitoring is tested to confirm that the proposed system can judge insert fracture, BUE, chipping, and wear. The results demonstrate that the standard deviation of the chipping and amount of wear accounts for 0.67% and 0.62%, of the average value, respectively, thus confirming the stability of system operation.

## 1. Introduction

**Figure 1:**
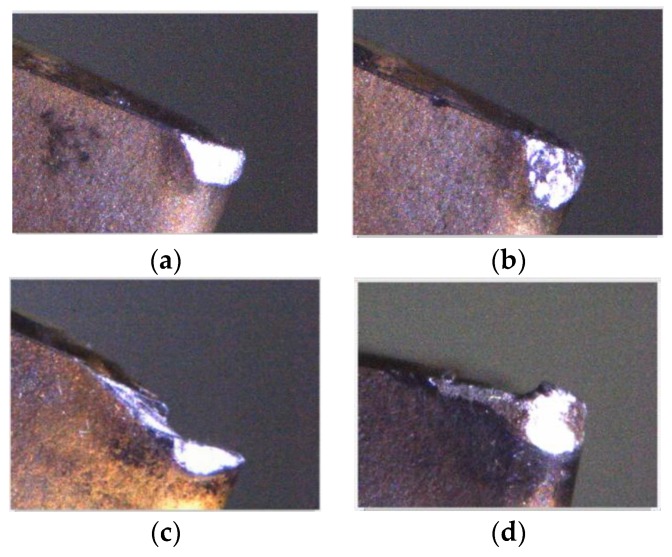
Four insert status forms. (**a**) Flank wear; (**b**) Chipping; (**c**) Fracture; (**d**) BUE.

The quality of mechanical parts is dependent on the accuracy of the machining tools and the abrasion conditions of cutting tools. For instance, Fernández-Valdivielso et al. [1] analyzed the effects of geometrical features of inserts on workpiece surface integrity and developed an indirect method for determining the geometrical features of inserts that achieve the best performance in machining difficult-to-cut alloys. Pereira et al. [2] considered the abrasion conditions on the interface between an insert and a workpiece, and proposed a coolant structure that combines cryogenic cooling and the minimum quantity of lubrication to improve tool life and workpiece surface integrity. Thus, to improve the quality of products, mechanical part manufacturers must be aware of the service behaviors of cutting tools in the actual machining process, as determined from the on-machine cutting tool condition monitoring system, to be able to analyze tool life and decide whether the cutting tool needs to be changed [3,4]. The insert wear formation mechanism in the turning process comprises abrasion, diffusion, oxidation, fatigue, and adhesion wear. As shown in Figure 1, flank wear, chipping, fracture, and built-up edge (BUE) occur most frequently in general cutting processes and are mostly concentrated at the tool tip and tool flank [5,6,7,8]. Therefore, these four conditions are classified in this study, and the insert condition is reviewed by visual inspection. Flank wear gradually occurred at the cutting insert owing to the erosion between the portions of the insert in contact with the workpiece. Excessive cutting force can usually lead to brittle fracture of a cutting insert. However, due to the high temperature at the contact area between the workpiece and the insert during the machining processes, the BUE (the phenomenon that the machined material builds up on the insert edge) occurs and it could break away from the insert edge and could carry a portion of material from the insert, thereby causing fracturing and chipping.

There are two types of insert condition inspections in turning processes: one is indirect inspection, where the external sensors feedback the analytical machine data [9,10], and the other is direct inspection, where the cutting tool status is measured [11,12]. Indirect inspection analyzes data to estimate the cutting tool status; some machine states are analyzed according to a reference, which means that the cutting status is systematically evaluated, thus replacing the judgment of experienced operators to reduce human errors and enhancing the ability of production automation [13]. For example, the cutting tool wear condition is analyzed based on the difference in the cutting noise or vibration [14,15], the cutting tool is monitored by measuring the changes in cutting temperature and cutting forces [16,17], and the cutting status is analyzed using the machine power or current variation signal [18]. All these methods use sensing signals for inspection analysis. Lately, indirect inspection by a charge-coupled device (CCD) camera has become popular. It analyzes the cutting tools by capturing the workpiece surface texture in images to determine whether the cutting tool is worn, judged according to the changes in the workpiece surface texture and surface roughness [19,20,21,22,23]. Some studies have focused on the fusion of multiple sensors and visual information of images for further tool status monitoring [24,25] or used different algorithm models for analysis to implement more accurate monitoring and evaluation [26,27,28,29]. According to the aforementioned references, the status of cutting tools can be obtained by analyzing machine information variations; however, such indirect inspection sometimes reduces the accuracy of the system under the effect of the external sensing environment [30]. Therefore, a direct inspection method is required to analyze the changes in the status of cutting tools.

Direct inspection analyzes the problems in machining by directly observing the practical situation of the cutting tool. Some documents use sound, light, or a probe to build a cutting tool model to observe the status of the cutting tool [25,31,32]. However, such measurement equipment is relatively complicated and unsuitable for onsite inspection. Another method uses a charge-coupled device (CCD) camera to capture tool images and analyze the status of cutting tools. There are two types of analysis regarding the status of cutting tools, one is to analyze the wear condition by outer contour and profile inspection [33], which is generally used to monitor the outer profile wear status to judge whether the cutting tool is still workable. In comparison with the indirect inspection methods that are used to analyze and inspect the surface texture of a machined workpiece to determine the tool status, the direct inspection method is to judge the status of the cutting tools by surface texture or surface roughness analysis of the tool edge after machining [34], which is applied for a more detailed inspection of the cutting tool and the machine states, as it provides detailed machining information. The general visual inspection of a CCD camera analyzes the different locations of a cutting tool, for example, some studies have implemented analysis according to crater wear [35,36], whereas others have implemented it according to the flank wear condition [37]. A majority of the status information regarding a cutting tool can be gathered by visual inspection; in other words, changes in the outer profile can be obtained from the images. Giusti et al. [38] proposed a visual inspection method for cutting tool wear, Rangwala and Dornfeld [39] proposed using a neural network to analyze wear status, and many scholars successively proposed other related inspection methods [26]. Regarding the methods for optimization of wear features, Kurada and Bradley [40] proposed using gradient operators to calculate texture features, where the wear region boundary feature search was calculated using an octagonal-shaped matrix, and the slope was established by the brightness difference and radial distance from the matrix center to determine the location of optimized wear features. The original image was smoothed during preprocessing to reduce the interference of irregularities. For feature calculation, the pixel value was converted by image thresholding to obtain the actual wear intensity and determine the change in wear amount. Yuan et al. [41] proposed a new filtering method to obtain average images and proposed a new edge detection method based on wavelet transform. When the wavelet function is selected, a new wavelet function is generated that describes the gray change of the image. In other words, noise interference can be avoided to obtain better edge features and the abrasion region, width, length, and center location of abrasion region can be measured. Wang et al. [42] proposed an image processing procedure, which is different from the traditional method based on constant thresholding. In this method, a rough-to-fine strategy is considered. First, the thresholding images are obtained for the search candidate’s wear bottom edge points. Then, the threshold-independent edge detection method, based on moment invariance, is used to determine the wear-edge. To shorten the computing time, a critical area is defined first, and only this area is taken as the region of interest in subsequent processes; thus, evading the threshold-dependent wear features detection method. Li et al. [43] used the pulse-couple neutral networks (PCNN) of bionics in cutting tool wear monitoring and used the spatial neighbor and similar gray clusters of pixels to segment the binary image of tool wear according to the condition that the gray intensity is higher than the body of the tool and background in the field of tool wear. Shahabi and Ratnam [44] used the external profile of the original image to test the alignment of the tool image and then used median filtering, morphological operations, and thresholding algorithms to reduce the system errors resulting from cutting tool misalignment, the presence of micro-dust particles, vibrations, and the intensity variations of ambient light. The aim was to determine the tool holder position and positioning error to ensure that cutting tool wear could be inspected without precision tool alignment. Pfeifer and Wiegers [45] used light source changes to determine wear-edge features under different light sources. While light changes can influence the shadow changes of the cutting tool wear-edge, the actual edge location does not vary with the light source. Thus, cutting tool wear image information under different light sources can be obtained using high-pass filter and thresholding images and the recurrent edge location can be obtained by overlapping to determine the location of a strong edge to filter out the misrecognition due to contaminants and reduce the effects of contaminants and shadow changes on the inspection system. Barreiro et al. [46] used different moments as descriptors to illustrate the tool wear images and then used a finite mixture MCLUST model to classify tool wear conditions into low-, medium-, and high-wear classes. Furthermore, the monitoring results were validated through the use of linear and quadratic discriminant analyses. Based on the image processing results of the cutting edge, Alegre et al. [47] developed a procedure to determine the time for tool replacement through the use of k-nearest neighbors and a multilayer neural network. D’Addona and Teti [48] used an image standardization process to obtain images with standard size and pixel density during cutting tests; then, the back-propagation neural network was optimized and used to estimate tool wear conditions with standardized cutting tool images.

Differing from existing research findings, this study analyzes insert statuses and uses fusion contour and texture inspection methods to build a more accurate evaluation and judgment system, which is applicable to on-machine automatic inspections and eliminates the environmental problems during inspection. A visual inspection system that can be used in CNC turning machine tools is constructed, which consists of a CCD camera and a lens for capturing insert images, a protection box to protect the photographic equipment, and a peripheral circuit and components, to avoid scrap splashes and cutting fluids during the cutting processes. The visual inspection system has a cleaning air tube, which jets air toward the insert to clean the surface of the inspected insert, thus, reducing the problems of subsequent image processing and increasing the accuracy of the insert condition judgment. The visual inspection system designed in this study has a surrounding light source and a fill-light for the tip of the insert to ensure that the insert condition can be analyzed under changing lighting conditions. When the light source is adjusted to determine the location of the blade and the tip of the insert, it is applicable to insert condition monitoring if onsite tool alignment is not accurate, thus enhancing the feasibility of image recognition in the machine. The light source intensity is adjusted and the insert image is captured under varying intensities for inspection analysis. The effect of any external environment changes on the insert condition monitoring result can be reduced and the system designed in this study can obtain accurate results in different environments. Image underexposure or solarization that generally result from changes in the insert condition are also improved. This study analyzes captured insert images with different features and the common insert conditions of an external turning tool, including fracture, BUE, chipping, and wear, can also be inspected. The analysis of the results can be quantized according to the texture feature distribution. This study conducts on-machine insert condition monitoring experiments with inserts in different states and the results show that the insert condition monitoring system designed in this study is applicable to computer numerical control (CNC) turning machine tools for correct and stable identification of insert fracture, BUE, chipping, and wear conditions. Contributions of this study therefore include

- development of an on-machine insert condition monitoring system that can be used to one-time identify the four insert conditions—fracture, BUE, chipping, and flank wear.
- development of a mountable visual system with different light sources to on-machine capture good-quality insert images that can be exactly analyzed under different lighting conditions.
- development of a contour and texture fusion inspection method to reduce environmental problems and to accurately identify insert conditions during inspection.

The structure of this paper is as follows. Section 2 describes the experimental system and related equipment used in this study, along with the hardware architecture design of the machine vision inspection system. Section 3 describes the insert image capture process designed in this study and the usage of the surrounding light source and fill-light for the insert tip. Section 4 describes the insert condition monitoring classification process designed in this study, including the insert outer profile construction, insert status region capture, and wear region judgment and calculation. Section 5 describes the experimental process and results of insert condition monitoring. The experiment on the on-machine insert condition monitoring by a CNC turning machine tool validates the feasibility and stability of this system. Section 6 summarizes this paper. 

## 2. Introduction to the Experimental System and Equipment

**Figure 2:**
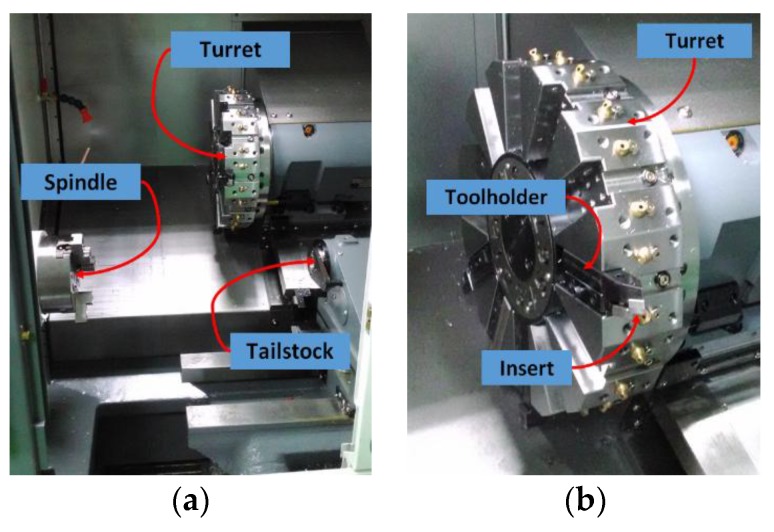
CNC turning machine tool for experiment. (**a**) Turning zone; (**b**) Turret structure.

**Figure 3:**
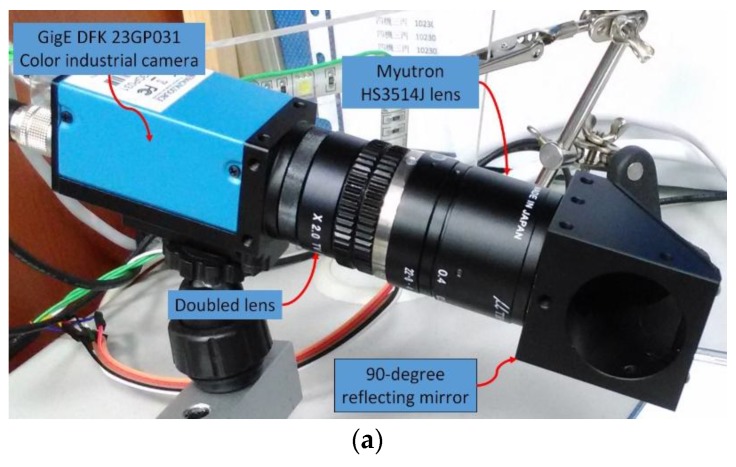

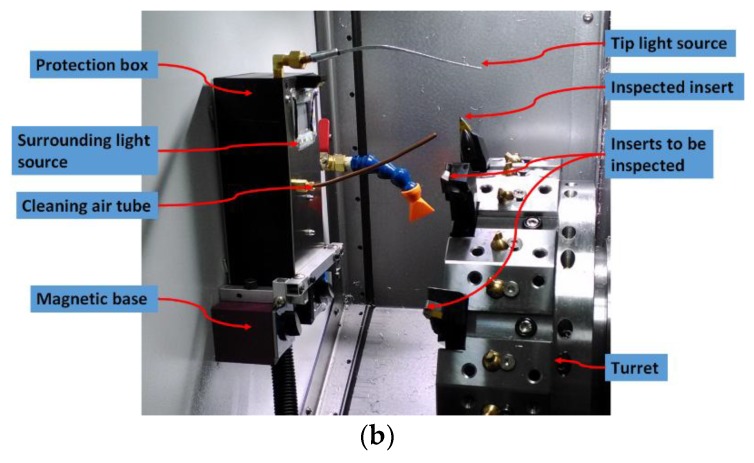
Visual inspection system mountable inside turning machine tools designed in this study. (**a**) Industrial camera and lens related components; (**b**) Visual inspection system protection box.

The CNC turning machine tool used in this study, shown in Figure 2, is tested using an external turning tool. The test external turning tool is mounted on the turning machine tool turret and the turning machine tool turret is moved by a computer numerical controller to the visual inspection system placed above the turning machine tool spindle for insert condition monitoring. Here, each insert position is adjusted by moving the turret such that the region of interest is focused in order to reduce the blurring of captured images. Moreover, during the period of experiments, the security door that is usually used to protect operators was closed so that the turning zone environment can reduce the influence from external environments. A GigE DFK 23GP031 color industrial camera, with an image resolution of 2592 × 1944 (15 fps), is used in this study. Figure 3a shows the camera hardware combination; the lens is a Myutron HS3514J CCTV lens, combined with a double lens to capture the feature image and the 90-degree reflecting mirror can adjust the angle of the camera. Due to the space constraints of the internal structure of the machine and considering the potential contamination resulting from the actual machining environment, this study designs a visual inspection system that can be mounted in turning machine tools, as shown in Figure 3b. The protection box for the camera hardware, as shown in Figure 3a, prevents the cutting scrap in the machine from splashing, thus, reducing the contamination of cutting fluid on the lens. To capture sharp insert images, the cleaning air tube jets air toward the insert for cleaning. The protection box is equipped with a surrounding LED light source with adjustable brightness and is covered with epoxy resin for protection. The protection box extends the fill-light for the tip of the insert to be inspected (tip light source). Two magnetic bases are set up at the protection box base to fix the protection box in the machine tool for on-machine insert condition monitoring. 

## 3. Insert Image Capture Process 

**Figure 4:**
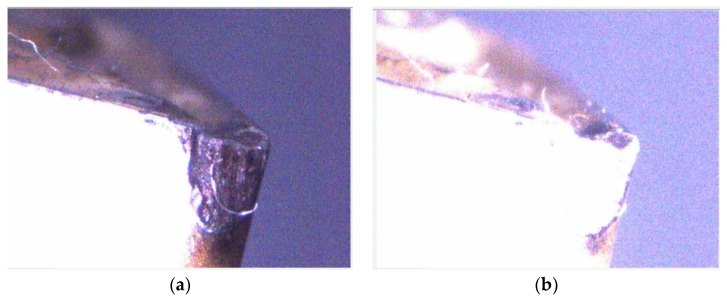
Captured flank and insert exposure images. (**a**) Flank exposure image; (**b**) Insert exposure image.

**Figure 5:**
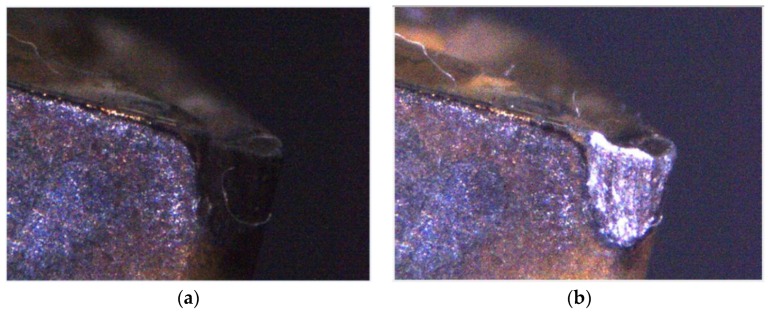
Captured flank and tip feature images. (**a**) Flank feature image; (**b**) Tip feature image.

During image capture, the fill-light is used for the inspected insert and the light source intensity is changed to ensure that different inserts have appropriate feature strength. This study uses two light sources in different positions as shown in Figure 3b. In terms of the surrounding light source, a strong light irradiates the test insert to obtain its surface shape and area features. In terms of the fill-light for the tip of an insert, the tip status feature is enhanced to facilitate later processing and analysis of the captured image. In the insert image capture process, as designed in this study, the insert is shot under a high-strength surrounding light source to capture the tool flank exposure image, as shown in Figure 4a. Then the insert image is captured using a high-strength surrounding light source and fill-light for the insert tip, as shown in Figure 4b. Here, the exposure images can be sequentially used to confirm the insert position, enhance geometry features, and strengthen wear features. The featured images are captured after the exposure image capture. First, the fill-light for the tip of an insert is closed and the surrounding light source intensity is adjusted to obtain appropriate flank feature images, as shown in Figure 5a, and then the intensity of the fill-light for the tip of an insert is adjusted to obtain appropriate tip feature images, as shown in Figure 5b. Here, the adjustment of light source intensity is automatically performed depending on the average thresholding value of the captured images. Referring to the exposure images, as shown in Figure 4, the feature images are used to analyze different insert conditions and can be utilized in the classification process of the insert conditions, including insert profile construction, status region capture, and wear judgment and calculation.

## 4. Insert Condition Monitoring Classification Process 

### 4.1. Insert Outer Profile Construction 

**Figure 6:**
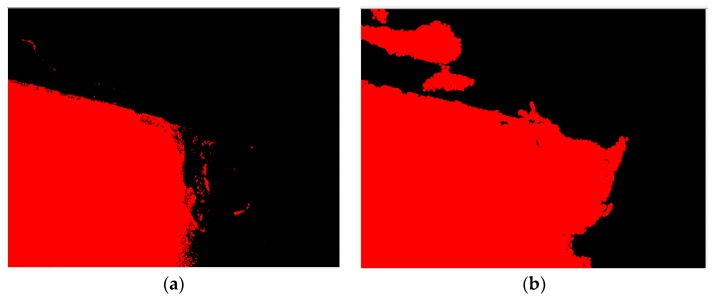
Thresholding operation result of captured exposure insert grayscale image. (**a**) Flank exposure thresholding image; (**b**) Insert exposure thresholding image.

**Figure 7:**
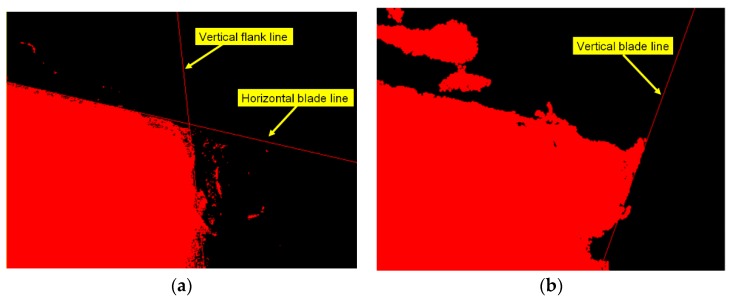
Lines of thresholding images. (**a**) Vertical flank line and horizontal blade line of flank profile feature; (**b**) Vertical blade line in insert exposure image.

**Figure 8:**
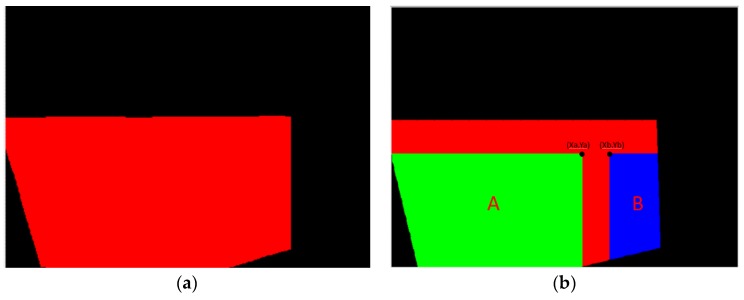
Completed insert outer profile thresholding images and block division. (**a**) Completed insert outer profile thresholding image; (**b**) Insert outer profile block division.

**Figure 9:**
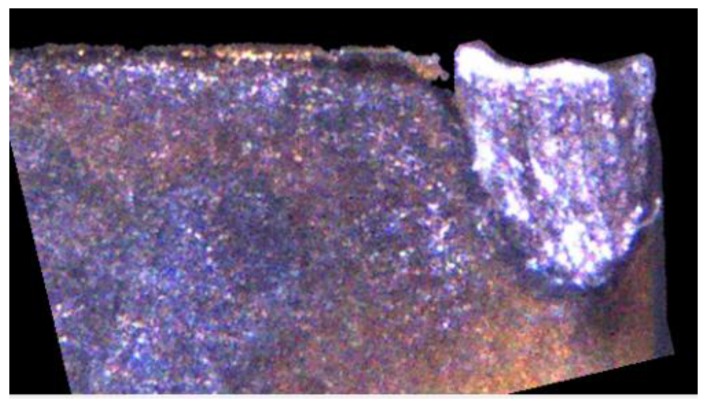
Trimmed insert outer profile image.

First, the flank profile feature is determined using the flank exposure image in Figure 4a. This study uses grayscale image thresholding to determine the flank profile feature, as shown in Figure 6a. Similarly, the insert profile feature in Figure 4b and grayscale image thresholding are used to determine the insert profile feature, as shown in Figure 6b. Here, the thresholding value is 250. The lines in the thresholding images in Figure 6 are determined using straight-line Hough transform, as shown in Figure 7. The flank profile exposure thresholding images determine the vertical flank line and horizontal blade line, while the insert profile exposure thresholding images determine the vertical blade line. The thresholding image can be trimmed and rotated along the horizontal blade line (Figure 7a) and vertical blade line (Figure 7b) in Figure 7 to construct a complete insert outer profile thresholding image, as shown in Figure 8a. According to the vertical flank line in Figure 7a, the complete insert outer profile thresholding image is divided into two blocks, as shown in Figure 8b: tip front-end underside (block B) and insert backend underside (block A) for subsequent insert condition feature recognition. Figure 9 shows the results of the trimmed insert images by referring to the completed insert outer profile thresholding image.

### 4.2. Insert Status Region Capture 

**Figure 10:**
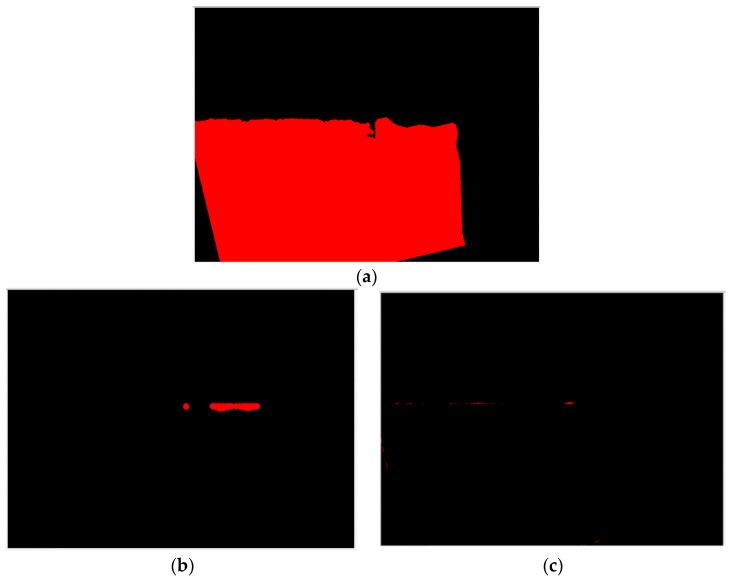
Judgment of insert fracture and BUE statuses. (**a**) Insert thresholding image; (**b**) Insert fracture zone; (**c**) Insert BUE zone.

The horizontal blade line in Figure 7a can be used to judge whether the insert has a fracture or BUE. The insert thresholding image in Figure 10a is obtained after the grayscale image thresholding process of Figure 9. Here, the erosion and dilation operations with the 11 × 11 diamond-shaped structuring element are used to clear the geometry features. The insert thresholding image is segmented along the horizontal blade line to obtain the insert fracture zone in Figure 10b and the insert BUE zone in Figure 10c, where the pixel areas of the fracture zone and BUE zone are calculated to judge the insert fracture or BUE status. 

**Figure 11:**
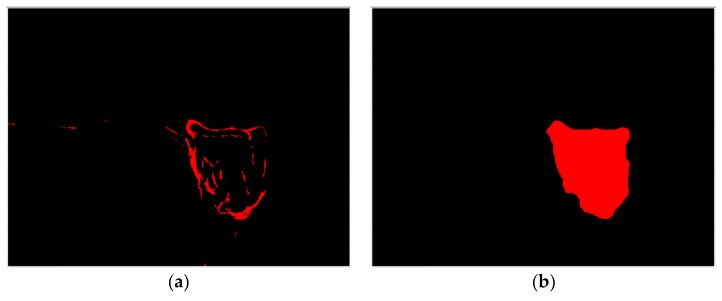

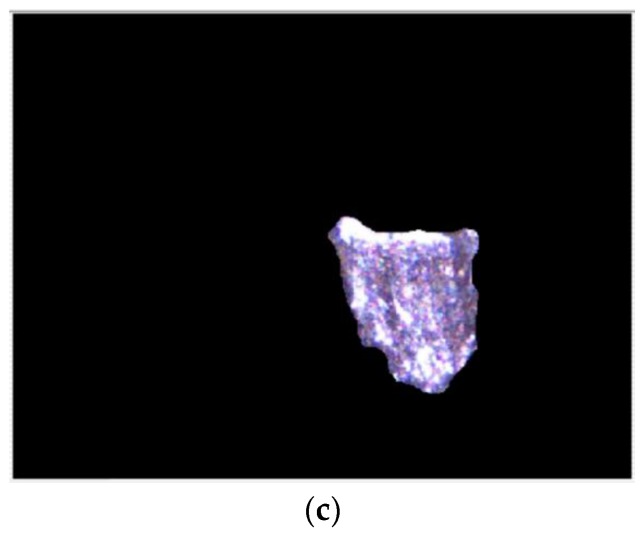
Actual image of trimmed insert wear region. (**a**) Flank wear feature zone; (**b**) Range of flank wear zone; (**c**) Flank wear zone image.

If the insert condition, as identified by the insert condition monitoring system designed in this study, is not classified as fracture or BUE, the flank wear judgment process begins. First, the grayscale transformation is implemented for the trimmed insert outer profile image in Figure 9. This study uses the average image RGB values for grayscale processing. After the insert outer profile image is converted into a grayscale image, the Sobel operator is used for insert edge detection to obtain a good insert edge feature. The insert outer profile blocks are then segmented, as shown in Figure 8b, and the lower region at the backend of the insert (block A) is removed to segment the location of the flank wear feature, as shown in Figure 11a. To facilitate the trimming of the flank wear part for subsequent judgment and calculation, the computation for noise removal, contrast stretching process, erosion, and dilation operations are implemented, as shown in Figure 9, and the flank wear zone image is obtained, as shown in Figure 11b. Here, the 3 × 3 box-pattern low-pass filter and the 21 × 21 box-pattern median filter are used for noise suppression. Finally, the trimmed operation is implemented for the insert outer profile image in Figure 9 according to Figure 11b and the flank wear zone image in Figure 11c is obtained.

### 4.3. Wear Region Judgment and Calculation 

**Figure 12:**
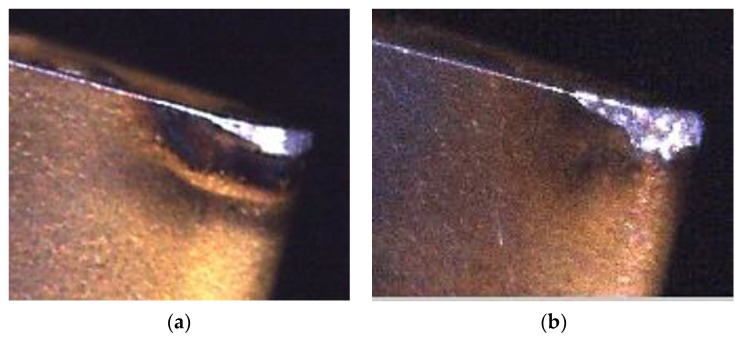
Comparison between flank wear and chipping wear. (**a**) Flank wear; (**b**) Chipping wear.

**Figure 13:**
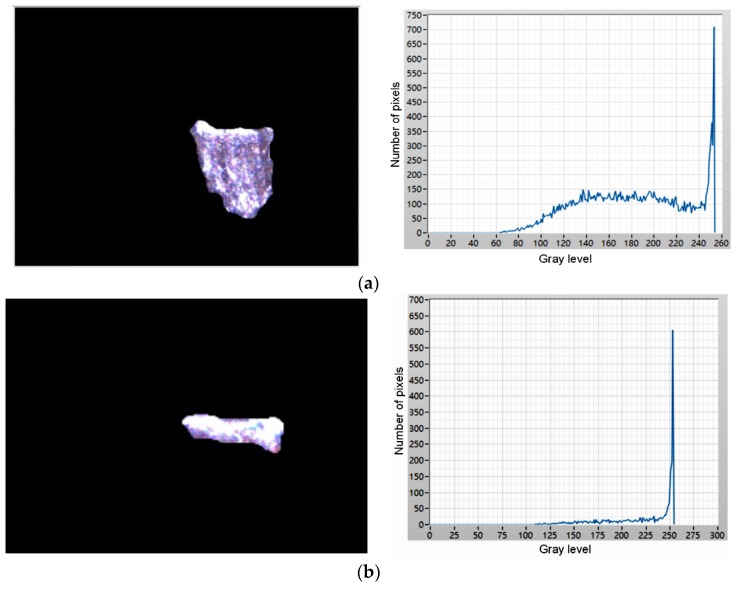
Histogram of grayscale image. (**a**) Grayscale image of Figure 11c; (**b**) Grayscale image of flank wear.

**Figure 14:**
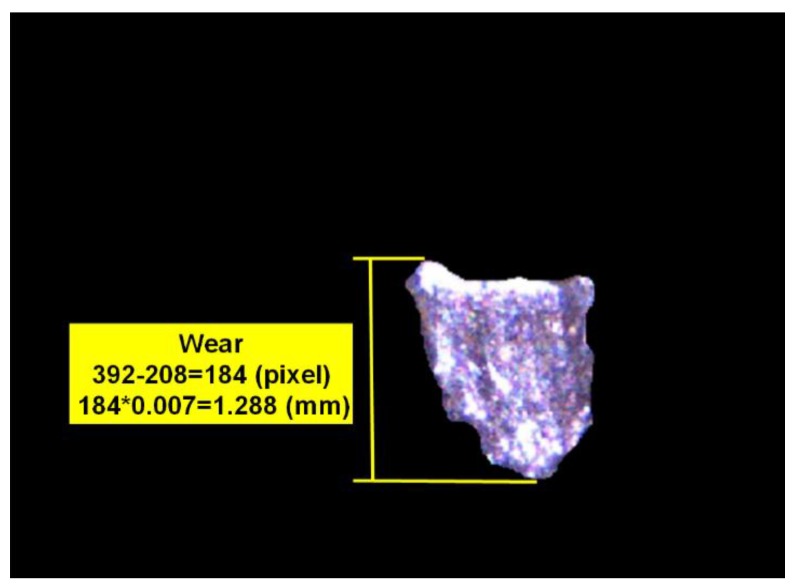
Wear amount result of wear region image.

The flank wear or chipping wear status can be classified according to the trimmed flank wear zone image, as shown in Figure 11c. Figure 12 shows that there is a significant difference between the flank wear and chipping wear. The flank wear is the tear resulting from the rub between the cutting blade and workpiece in the machining process, thus, the flank wear surface features are mostly continuous and even. However, as chipping wear is the tip breakage resulting from abnormal machining processes, the chipping surface is relatively rough. This study analyzes the continuity of surface features for the actual image of a wear region to identify the insert wear region as flank or chipping wear, as shown in Figure 11c. The grayscale value histogram of all pixels can be obtained after Figure 11c is converted into a grayscale image, as shown in Figure 13a. The number of pixels is obviously larger than the pixel grayscale value histogram distribution of the flank wear image, as shown in Figure 13b. Therefore, the number of pixels larger than the preset threshold value is divided by the calculated value percentage of the number of pixels of the overall wear region to identify the wear region as flank or chipping wear. In other words, the percentage (chipping rate) of the number of pixels larger than the preset threshold value to the number of pixels of the overall wear region is taken as the basis of judgment. Moreover, this study uses the length of the pixels of the upper and lower boundaries of the wear region image to calculate the wear amount. The pixel unit is converted using the wear region image, as shown in Figure 14, where the conversion length of the wear region image pixels is 0.007 mm and the length in the pixels of the upper and lower boundaries of wear region image is 184 pixels, thus, the converted wear amount is 1.288 mm. 

## 5. Experiment Monitoring Insert Condition 

**Figure 15:**
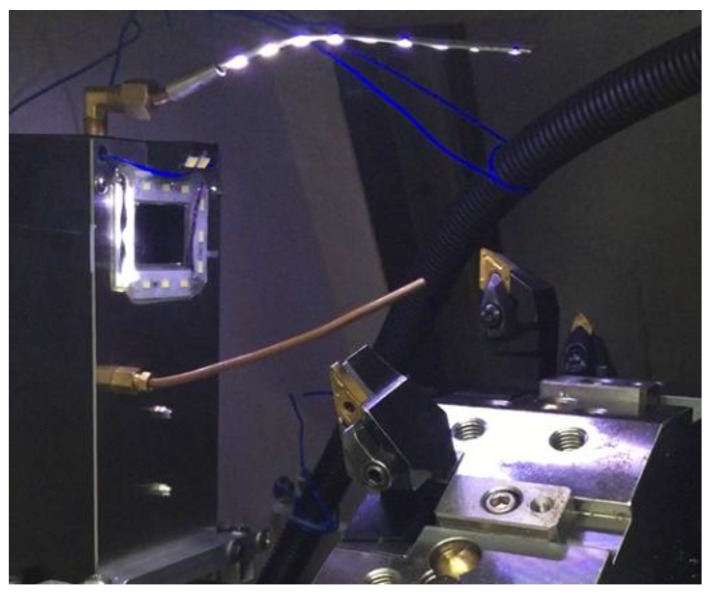
Experimental system setup for insert condition monitoring.

**Figure 16:**
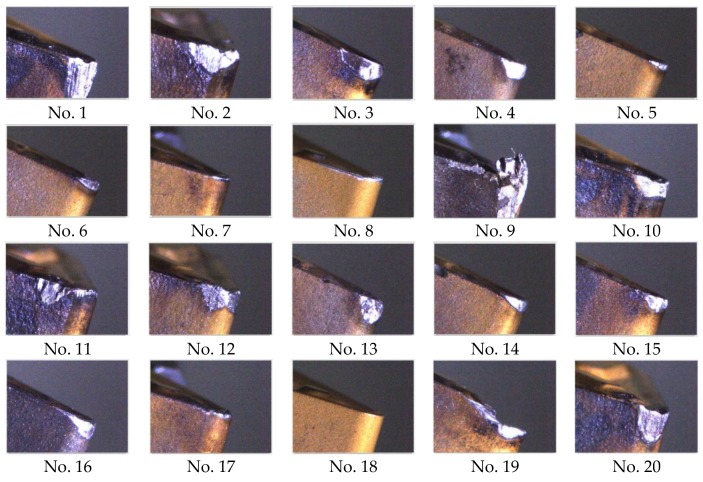
Captured images of different insert conditions (corresponding to insert numbers in Table 1).

To validate the feasibility of the on-machine insert condition monitoring system proposed in this study, the visual inspection system mounted on the turning machine tool for insert condition monitoring experiments is shown in Figure 15. This study uses twenty used inserts in various states for experimentation and the results are presented in Table 1 and Figure 16. Here, the used inserts were collected after turning with cutting speed (130–150 m/min), cutting feed rate (0.2–0.3 mm/rev), and depth-of-cut (2–3 mm). The workpiece material is medium carbon steel and the insert material is tungsten carbide. The laptop computer with an Intel Core i7-4720HQ, 2.6-GHz CPU, and 64-bit Microsoft Windows 10 operating system was utilized to implement the whole system so that the time required for each monitoring task is approximately 13 s in which 2.75 s, on average, are required for the identification of insert conditions. To further reduce the time required for each monitoring task, a computer with a faster CPU could be used to implement the system. Table 1 shows the judgment results of the inserts in different states. The chipping rate is set at 50% for monitoring and the wear amount is set as 0.3 mm for identifying over-wear. Based on the results, the system developed in this study can correctly identify the various insert conditions of the test inserts. Table 1 presents three types of BUE inserts, where two of them have slight BUE. Thus, it can be said that this study identifies the BUE status accurately according to the preset threshold of BUE.

The insert condition monitoring system can identify different insert conditions and its operational stability is a key point of evaluation. Due to the changing external environment and light source intensity, there will be different results for insert condition tests and calculations. This study repeatedly tests the same insert to validate the stability of the insert condition monitoring system and the experimental results are shown in Table 2, where the wear amount of the insert wear region is calculated for comparison analysis. The experiment is repeated 10 times, the chipping rate and wear amount of each experiment are recorded, and system stability is checked using the calculated mean value and standard deviation. The experimental results show that the chipping rate analysis has large standard deviation, which signifies that there is a large variation in the results. The chipping rate is calculated according to the pixel grayscale value histogram distribution of the flank wear zone image, even though the algorithm and light source system operating procedure are identical, each moment of image capture is affected by the light source change and the grayscale value histogram distribution of the wear images changes. Despite all this, the standard deviation of the chipping rate is only 0.67% of the average value and the stability of the chipping rate calculation of this insert condition monitoring system can be calculated. In terms of wear amount results, the standard deviation of wear amount is only 0.62% of the average value, in other words, the standard deviation is lower than two pixels. Hence, the stability of this insert condition monitoring system in calculating wear amount can be calculated. Therefore, the aforementioned experimental results can be used to validate the feasibility and stability of the insert condition monitoring system and calculation method designed in this study.

This study developed an on-machine insert condition monitoring system to identify four external turning tool insert conditions; fracture, BUE, chipping, and flank wear. The experimental results demonstrate that the developed monitoring system can successfully identify the four insert conditions. Moreover, as shown in Figure 16, the developed system can be used for identifying the insert conditions when it is difficult to measure the wear amount precisely using standard wear measurement methods. However, because the view angle of the developed visual inspection system that is mounted inside the turning machine tools is different from the view angle of standard wear measurement devices, the calculated wear amount, which is used to indicate the degree of wear conditions, cannot be compared with the measurement results obtained using standard wear measurement devices.

## 6. Conclusions

The status of cutting tools used in the cutting processes of machine tools will obviously influence the manufacturing quality of machine parts. Therefore, this study develops an on-machine insert condition monitoring system for the turning tool insert of CNC turning machine tools and uses the machine vision method to inspect the common flank wear, chipping, fracture, and BUE statuses of turning tool inserts. This study differs from the existing research methods and outcomes as it fuses the machine vision method with contour and texture inspections to analyze the insert status. This eliminates the environmental problems in the insert inspection process to build a more accurate on-machine turning tool insert condition monitoring system. 

To fix the CCD camera and lens in the CNC turning machine tool to carry out the on-machine insert condition visual inspection process, a visual inspection system with a protection box, cleaning air tube, and two light sources is designed. The protection box can avoid the scrap splash and contamination of cutting fluid on the lens during the cutting processes, while the cleaning air tube jets air toward the insert to clean off surface contaminants. A surrounding light source and a fill-light for the tip of an insert with variable light intensities are employed to analyze the effect of change in lighting conditions on the visual inspection of the insert status. In the insert image capture process, the intensity of the surrounding light source and fill-light is changed to ensure that the test insert has appropriate feature strength. The surrounding light source uses strong light to irradiate the insert surface to obtain the surface shape and area features, while the fill-light enhances the tip status feature to facilitate subsequent captured image processing and analysis. An insert condition monitoring classification process designed in this study includes insert outer profile construction, insert status region capture, and wear region judgment and calculation. The insert outer profile construction uses the exposure image to determine the outer profile feature, and then the vertical flank line, horizontal blade line, and vertical blade line are established according to this outer profile feature. The insert image can be trimmed for subsequent insert condition feature recognition. In terms of insert status region capture, the insert fracture zone and BUE zone are identified according to the outer profile feature lines and the insert outer profile image is trimmed to obtain the actual image of the insert wear region. For insert wear region judgment and calculation, the flank wear or chipping wear is identified based on the grayscale value histogram of all pixels of the trimmed flank wear zone image. The wear amount is calculated using the pixel length of the upper and lower boundaries of the wear region image, which are used as the reference index to identify the normal wear or over-wear status of the insert. Finally, inserts in different states are used for on-machine insert condition monitoring experimentation to confirm that the system designed in this study can identify insert fracture, BUE, chipping, and wear statuses. In addition, as the changes in external environment and light source sometimes influence the image processing result, the operational stability of the on-machine insert condition monitoring system is tested in this study. The experiment is conducted repeatedly and the average value and standard deviation of the chipping rate and wear amount in the experimental results are recorded as the basis for evaluating the operational stability of the system. The experimental results show that the light source variation does influence the calculation of chipping rate and wear amount. The standard deviation of the chipping rate is only 0.67% of the average value, while the standard deviation of wear amount is 0.62% of the average value (standard deviation lower than 2 pixels), thus validating the stability of system operation. 

## Figures and Tables

**Table 1:** Experimental results of condition monitoring of different inserts.

No.	Chipping Rate (%)	Wear Amount (mm)	Status Determination	No.	Chipping Rate (%)	Wear Amount (mm)	Status Determination
1	45.76	1.530	over-wear	11	62.47	0.651	chipping
2	0.00	0.000	BUE	12	0.00	0.000	BUE
3	35.14	0.735	over-wear	13	52.42	0.875	chipping
4	20.65	0.658	over-wear	14	19.26	0.427	over-wear
5	26.04	0.238	normal wear	15	37.09	0.532	over-wear
6	33.75	0.287	normal wear	16	31.35	0.903	over-wear
7	12.20	0.105	normal wear	17	29.48	0.161	normal wear
8	26.34	0.252	normal wear	18	0.00	0.000	normal wear
9	0.00	0.000	BUE	19	0.00	0.000	fracture
10	29.26	0.686	over-wear	20	63.06	1.250	chipping

**Table 2:** Experimental results of computational stability of insert wear.

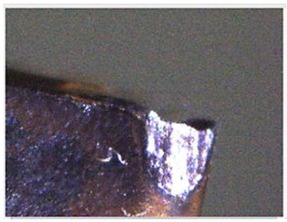	**No.**	**Chipping Rate (%)**	**Wear Amount (mm)**
	1	52.886	1.302
	2	52.993	1.288
	3	52.922	1.288
	4	52.831	1.302
	5	53.335	1.288
	6	53.003	1.288
	7	52.820	1.302
	8	52.746	1.295
	9	51.878	1.281
	10	52.760	1.302
	Average value	52.817	1.294
	Standard deviation	0.352	0.008
